# Refractory gastric ulcer bleeding responsive to long-term octreotide

**DOI:** 10.1177/2050313X231164856

**Published:** 2023-04-06

**Authors:** Humzah Iqbal, Dashmeet Maharaj, Hunza Chaudhry, Alakh Gulati, Marina Roytman

**Affiliations:** 1 Department of Internal Medicine, University of California, San Francisco, Fresno, CA, USA; 2 Department of Gastroenterology and Hepatology, University of California, San Francisco, Fresno, CA, USA

**Keywords:** Peptic ulcer disease, octreotide, refractory gastrointestinal bleed, endoscopic hemostasis

## Abstract

Upper gastrointestinal tract bleeding is a common condition that can cause hemodynamic instability and death if left untreated. Endoscopic hemostasis is often successful; however, some patients may develop refractory bleeding. Pharmacologic management with octreotide is beneficial in patients with variceal bleeding and has been shown in some studies to be effective in refractory bleeding due to angiodysplasia. There is a paucity of literature regarding the usage of long-term octreotide in refractory bleeding secondary to a peptic ulcer. We present a case of a bleeding gastric ulcer that was refractory to endoscopic management but responsive to long-term octreotide therapy.

## Introduction

Upper gastrointestinal tract bleeding (UGIB) is a potentially life-threatening condition that can have a mortality rate up to 10% and accounts for more than 300,000 hospital admissions per year in the United States.^1^ UGIBs can occur anywhere in the upper gastrointestinal (GI) tract and are most commonly a result of peptic ulcer disease (PUD), which can account for as much as 60% of all acute UGIBs.^2^ PUD is usually caused by infection with *Helicobacter pylori*. Initial management is confirming the presence of bacteria with a urea breath test or stool antigen test, followed by eradication with a proton pump inhibitor (PPI) plus antibiotics. In patients who develop an acute UGIB due to PUD, endoscopic management along with intravenous (IV) PPI administration is the gold standard of treatment, and meta-analyses have demonstrated a decrease in re-bleeding and mortality with this approach. In some rare cases, patients may develop refractory bleeding despite optimal treatment, warranting repeat endoscopic management, surgery, or embolization of the bleed.^1,3^ However, there is a paucity of literature regarding guideline-based management if these approaches fail. Some studies suggest that octreotide, a somatostatin analogue, can be used in refractory UGIB of unknown origin.^4^ Octreotide is used to control bleeding secondary to esophageal varices, and some studies have demonstrated that it can be effective in UGIBs caused by angiodysplasia.^5,6^ We present a rare case of a refractory UGIB resulting from a peptic ulcer that was successfully treated with long-term octreotide.

## Case presentation

**Figure 1.**
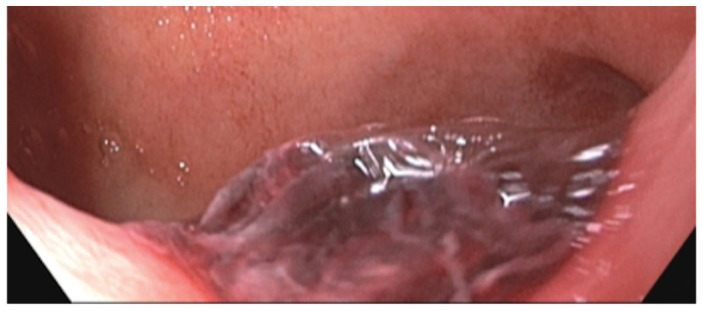
Peptic ulcer visualized at the pylorus on first EGD.

A 58-year-old male with a past medical history of diabetes mellitus and end-stage renal disease (ESRD) was admitted to the hospital with 2 weeks of intermittent melena. Laboratory results were significant for hemoglobin of 6.1 g/dL which was decreased from his baseline hemoglobin of 8–9 g/dL, iron of 22 ug/dL, international normalized ratio (INR) of 1.1, prothrombin (PT) time of 13 s, and activated partial thromboplastin (aPTT) time of 33 s. The patient was not on any anti-platelet agents or anticoagulation. Esophagogastroduodenoscopy (EGD) showed one 8-mm non-obstructing cratered gastric ulcer with an adherent clot (Forrest class IIb) found at the pylorus (Figure 1), and hemostasis was achieved with epinephrine and a bipolar probe with no other sites of bleeding. The patient was started on an IV PPI infusion and quadruple therapy for *H. pylori*, as well as daily ferrous sulfate for iron deficiency.

**Figure 2.**
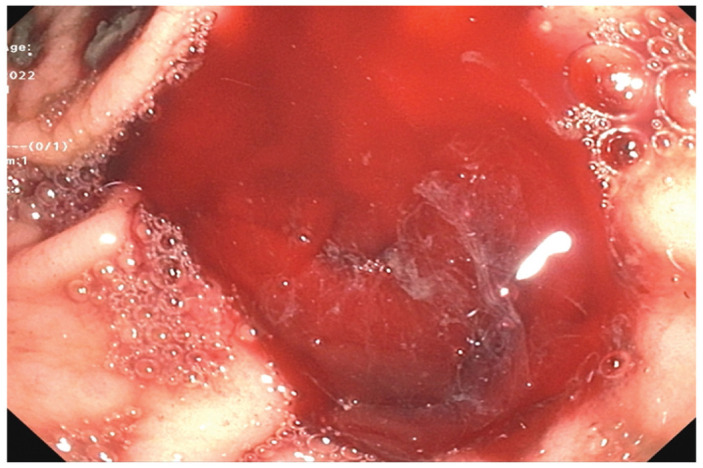
Fourth EGD showing recurrence of active gastric bleeding.

**Figure 3.**
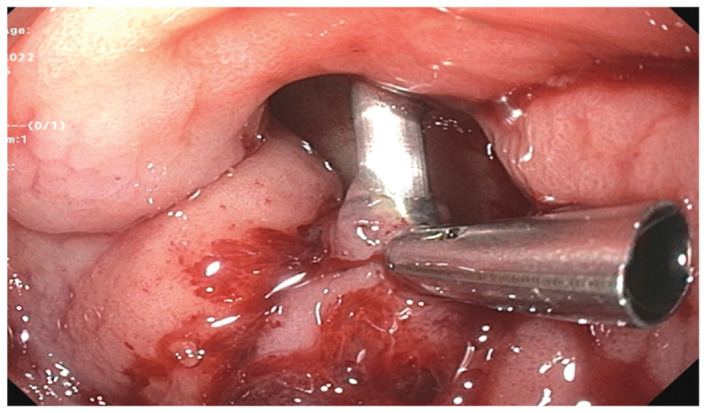
Application of hemostatic clips as part of the multi-modal management of the bleeding peptic ulcer.

The patient underwent two more EGDs due to recurrent bleeding with decreases in hemoglobin, which demonstrated active bleeding in the gastric antrum. Hemostasis was achieved with epinephrine, bipolar probe, hemostatic clip, and hemospray. Despite these interventions and continuation of IV PPI, the patient again developed recurrence of melena and an acute drop in hemoglobin to 6.4 g/dL. A computed tomography (CT) angiogram was obtained for potential embolization, which showed no signs of active bleeding, hence surgical evaluation was recommended. The patient was evaluated and determined to not be an adequate surgical candidate due to underlying comorbidities and poor functional status. He was continued on supportive treatment with stable vital signs despite further drops in hemoglobin to as low as 5.5 g/dL and multiple blood transfusions, receiving a total of 12 units throughout his hospital course. A fourth EGD was performed which showed similar findings as prior and hemostasis was achieved as previously (Figures 2 and 3).

The patient developed further melena and another drop in hemoglobin, which prompted initiation of a continuous infusion of octreotide for 72 h followed by 50 mg subcutaneous injections three times per day for 10 days. Hemoglobin increased back to his baseline of 8–9 g/dL over the next 2 weeks without transfusions. The patient was discharged with oral PPI and scheduled for repeat EGD in 3 months for evaluation of the ulcer.

## Discussion

UGIB is a potentially life-threatening condition, with mortality rates as high as 10%. While UGIBs are most commonly caused by PUD, they may also be sequelae of other conditions such as ESRD.^7^ ESRD increases the risk of bleeding, need for transfusion, re-bleeding rates, duration of stay, and mortality, and is thought to contribute to UGIB primarily due to uremic platelet dysfunction and increased risk for vascular malformations.^8^*H. pylori* infection can lead to PUD by invading the gastric mucosa and inducing inflammatory infiltration. Aspirin or non-steroidal anti-inflammatory drugs (NSAIDs) contribute to PUD by causing local erosion of the epithelium and exposing it to the acidic contents of the lumen, while also decreasing protective prostaglandin formation. Studies have shown mixed results; however, a meta-analysis found that there is insufficient evidence to determine whether *H. pylori* causes an increased UGIB bleeding risk in patients who take aspirin or NSAIDs.^9^

Most cases of GI bleeding are effectively treated with endoscopic intervention, which is generally successful and leads to a decrease in blood transfusions and length of hospital stay.^10^ Our patient underwent repeated endoscopic hemostasis for his bleeding ulcer without technical difficulty; however, he developed recurrent UGIB, which can be seen in 24% of patients who are at high risk for bleeding.^1^ IV PPI was administered in addition to endoscopic management, which can decrease the recurrence rate by as much as 10%.^11^ Repeat endoscopy is warranted in cases of recurrent bleeding, and patients usually achieve long-term hemostasis after this step in management. Despite receiving a total of four endoscopies in a 3-week period with multimodal hemostasis, our patient had a unique condition of intractable UGIB.

Octreotide is an analogue to the hormone somatostatin, which increases platelet aggregation, decreases splanchnic and gastroduodenal blood flow, and decreases gastric acid and pepsin secretion.^12^ These effects work synergistically to reduce the risk, severity, and duration of GI bleeds, particularly variceal bleeds. Octreotide may also be used in refractory cases as the primary therapy for GI bleeds and is often effective due to its alternate mechanism of action compared to traditional therapy. Most of the current literature regarding octreotide for refractory non-variceal bleeds involves vascular malformations or the presence of a left ventricular assist device (LVAD), conditions with well-known associations with GI bleeding. A systematic review by Brown et al.^13^ demonstrated a significant response to octreotide therapy in patients with refractory UGIB due to angiodysplasia. Octreotide use in these patient populations is associated with a decrease in hospital stay duration and overall cost of care.^6^

The dosage of octreotide in other reported cases was an initial infusion of 50 mg/h for 3–5 days, followed by 50–100 mg subcutaneous injections three times per day. The duration of octreotide therapy varies, with some studies demonstrating the usage of long-term or long-acting octreotide as an effective treatment modality.^14^ One study suggests that 1 year may be sufficient for controlling chronic bleeding from GI angiodysplasia.^15^ Despite its efficacy in multiple causes of refractory GI bleeding, rarely, long-term octreotide been reported to treat a refractory peptic ulcer bleed. In other reported cases, long-term octreotide was initiated for refractory ulcers only after surgical management had been ruled out, and patients achieved successful hemostasis with this regimen as seen in our patient. Octreotide should be considered as an adjunct or primary therapy in patients with non-variceal UGIB that has failed standard therapy in order to reduce associated morbidity and mortality.

## Conclusion

Octreotide is a commonly used drug agent to treat UGIB due to bleeding varices. Long-term octreotide is poorly studied in other causes of UGIB, with most studies reporting its use in bleeding that is secondary to angiodysplasia. We present a case of an intractable bleeding peptic ulcer that was successfully treated with long-term octreotide.
